# Detection of cytokeratin-19 mRNA-positive cells in the peripheral blood and bone marrow of patients with operable breast cancer

**DOI:** 10.1038/sj.bjc.6605183

**Published:** 2009-07-21

**Authors:** A Daskalaki, S Agelaki, M Perraki, S Apostolaki, N Xenidis, E Stathopoulos, E Kontopodis, D Hatzidaki, D Mavroudis, V Georgoulias

**Affiliations:** 1 Department of Pathology, University General Hospital of Heraklion, Crete, Greece; 2 Laboratory of Tumor Cell Biology, School of Medicine, University of Crete, Heraklion, Greece; 3 Department of Medical Oncology, University Hospital of Heraklion, Crete, Greece

**Keywords:** breast cancer, circulating tumour cells, CK-19, micrometastasis, disseminated tumour cells

## Abstract

**Background:**

To compare detection rates and evaluate the clinical relevance of cytokeratin-19 (CK-19) mRNA-positive cells in the peripheral blood (circulating tumour cells, CTCs) and bone marrow (disseminated tumour cells; DTCs) of patients with early breast cancer.

**Methods:**

Paired samples of peripheral blood and bone marrow were obtained from 165 patients with stage I–II breast cancer before the initiation of adjuvant chemotherapy. In 84 patients, paired blood and bone marrow samples were also available after chemotherapy. The detection of CK-19 mRNA-positive CTCs and DTCs was assessed by real-time PCR.

**Results:**

CK-19 mRNA-positive CTCs and DTCs were detected in 55.2 and 57.6% of patients before chemotherapy, respectively. After chemotherapy, CTCs and DTCs were identified in 44 (52.4%) and 43 (51.2%) of the 84 patients, respectively. There was a 93.9% (McNemar; *P*=0.344) and 72.6% (McNemar; *P*=0.999) concordance between blood and bone marrow samples before and after chemotherapy, respectively. The detection of CK-19 mRNA-positive CTCs or DTCs before chemotherapy was associated with decreased overall survival (*P*=0.024 and *P*=0.015, respectively). In addition, their simultaneous detection was also associated with an increased incidence of disease-related death and decreased overall survival (*P*=0.016).

**Conclusions:**

The detection of CK-19 mRNA-positive CTCs using reverse transcription-PCR (RT–PCR) both before and after chemotherapy is correlated with the detection of CK-19 mRNA-positive DTCs in patients with early-stage breast cancer. The determination of the CTC status by RT–PCR conveys clinically relevant information that is not inferior to DTC status and, owing to the ease of sampling, warrants further evaluation as a tool for monitoring minimal residual disease.

Although adjuvant chemotherapy and hormone treatment have improved disease-free and overall survival (OS) (EBCTC Group, 2005), almost 30% of patients with node-negative breast cancer will present distant metastases and will die as a result of disseminated disease (EBCTC Group, 1998). This is due to early tumour-cell dissemination through the lymphatic or the hematogenous vasculature (Dowlatshahi *et al*, 1997; Giatromanolaki *et al*, 2004; Stathopoulos *et al*, 2005). Indeed, epithelial tumour cells can be identified in bone marrow aspirates (disseminated tumour cells, DTCs) or in the peripheral blood (circulating tumour cells, CTCs) of otherwise metastases-free breast cancer patients using either immunohistochemical or molecular assays (Schoenfeld *et al*, 1997; Pantel *et al*, 1999; Slade *et al*, 1999).

The immunocytochemical detection of DTCs has been shown to be associated with decreased disease-free survival (DFS) and OS of patients with operable breast cancer (Mansi *et al*, 1999; Braun *et al*, 2000b; Wiedswang *et al*, 2003; Braun and Naume, 2005). We have reported earlier that the detection of CTCs before the initiation of adjuvant chemotherapy in patients with operable breast cancer, irrespective of the presence of axillary lymph node involvement (Stathopoulou *et al*, 2002; Wiedswang *et al*, 2003; Xenidis *et al*, 2006), or an oestrogen/progesterone or HER2 receptor expression (Ignatiadis *et al*, 2007), is also an independent factor associated with decreased DFS and OS.

The detection of DTCs and CTCs during both adjuvant and metastatic settings could be a useful tool for monitoring the efficacy of systemic treatment. It has been reported that adjuvant chemotherapy may eliminate DTCs (Braun *et al*, 2000a) or CTCs (Xenidis *et al*, 2003) in only 50% of patients. Moreover, the detection of CTCs during the administration of adjuvant tamoxifen is associated with decreased DFS and OS (Xenidis *et al*, 2007). Finally, Cristofanilli *et al* (2004) showed that the decrease in the absolute number of CTCs during chemotherapy in patients with metastatic breast cancer could be considered as an early event, indicating the efficacy of treatment. However, in patients with early breast cancer, who are asymptomatic, repeated and frequent bone marrow aspirations for the detection of DTCs may not be easily acceptable. Conversely, the use of blood for detecting CTCs is more convenient and could be easily acceptable by patients, thus representing a valuable alternative solution. Although some previous studies (Ismail *et al*, 2004; Pierga *et al*, 2004; Muller *et al*, 2005; Benoy *et al*, 2006; Wiedswang *et al*, 2006) have shown a correlation between the detection of DTCs and CTCs, this fact has not been unanimously accepted. This could be due to the fact that in most studies, the comparison of the detection of CTCs and DTCs was carried out using immunocytochemical assays, which, in general, have a lower sensitivity compared with molecular assays.

The main objective of this study was the direct comparison of the detection rate of cytokeratin-19 (CK-19) mRNA-positive CTCs and DTCs in paired samples of the peripheral blood and bone marrow obtained from patients with early breast cancer. The secondary objective was the evaluation of their prognostic significance in the same group of patients hypothesising that peripheral blood sampling could replace bone marrow aspiration as a prognostic tool in patients with early breast cancer.

## Patients and methods

### Patients

A study evaluating the clinical relevance of CK-19 mRNA-positive CTCs and/or DTCs in patients with early stage or metastatic breast cancer has been underway since 1996 at the Department of Medical Oncology at the University Hospital of Heraklion (Crete, Greece). Peripheral blood or bone marrow aspirates are obtained from patients who sign an informed consent, before the initiation of adjuvant or front-line treatment as part of their initial evaluation. In this study, we retrospectively identified a total of 165 patients who had all undergone adjuvant chemotherapy for stage I–II breast cancer from March 1999 to January 2004, and for whom paired blood and bone marrow samples before the initiation of any systemic treatment were available. In 162 (98.2%) of them, a blood sample was also available after the completion of treatment, whereas only 84 (50.9%) consented for a repeated bone marrow aspiration after adjuvant chemotherapy.

Every enrolled patient underwent a complete baseline diagnostic evaluation to exclude distant metastases before primary surgery, which consisted of chest X-rays, mammography, abdominal ultrasound and whole-body bone scan. Further imaging studies (computed tomography scans and magnetic resonance imaging) were carried out, if clinically indicated. Surgical treatment was either mastectomy or lumpectomy with axillary lymph node dissection. Radiation treatment was given to patients who underwent lumpectomy and to those with four or more axillary lymph nodes. All patients included in this study underwent adjuvant chemotherapy and most were treated in the context of research protocols of the Hellenic Oncology Research Group (HORG). Adjuvant chemotherapy consisted of either FEC (Fluoruracil, Epirubicin, Cyclophosphamide) or EC/T (Epirubicin, Cyclophosphamide/Taxotere), or classical CMF (Cyclophosphamide, Methotrexate, Fluoruracil) (details of the used chemotherapy regimens have been previously reported; Ignatiadis *et al*, 2007). Patients with HER2-positive tumours were not given adjuvant trastuzumab, because all were treated before the positive results from the adjuvant trastuzumab trials were reported. Patients with oestrogen receptor (ER)-positive and/or progesterone receptor (PR)-positive tumours were given 20 mg tamoxifen daily for 5 years; pre-menopausal women were also given luteinising hormone-releasing hormone analogues for 2 years.

Follow-up was conducted every 3 months during the first 2 years, every 6 months until 5 years after surgery and yearly thereafter, and it consisted of a clinical examination with routine laboratory studies (including tumour markers) and imaging studies with X-rays and abdominal ultrasound (computed tomography scans and magnetic resonance imaging were carried out, if clinically indicated). The administration of adjuvant chemotherapy and hormonal treatment was decided independently of the molecular detection of CTCs or DTCs. Moreover, during the follow-up period, clinicians were blinded for the results of CK-19 mRNA detection.

### Clinical samples

Peripheral blood (20 ml in EDTA) and bone marrow aspirations (3 ml in EDTA aspirated from the posterior iliac crest under local anaesthesia) were obtained before the initiation of adjuvant treatment (usually 3 or 4 weeks after primary surgery). Peripheral blood and bone marrow aspirates were also obtained after the completion of adjuvant chemotherapy from patients who gave their consent. All blood samples were obtained from vein puncture after the first 5 ml of blood was discarded; similarly, the initial 0.1–0.2 ml of bone marrow aspirate was discarded. This precaution was undertaken to avoid a contamination of the blood or bone marrow samples with epithelial cells from the skin during sample collection.

### RNA extraction and real-time PCR assay for CK-19 mRNA-positive cells

For this study, frozen RNA samples stored in the biobank of the laboratory of Tumour Cell Biology were used. Both peripheral blood and bone marrow samples had been processed for mononuclear cell isolation in room temperature within 2–4 h after sampling. Briefly, samples were diluted (vol/vol) with RNase-free, sterile 0.9% NaCl, and mononuclear cells (peripheral blood mononuclear cells and bone marrow mononuclear cells, respectively) were obtained by Ficoll-Hypaque (Sigma-Aldrich, St Louis, MO, USA) gradient density centrifugation at 1200 **g** for 30 min at 4 °C. Interface cells were obtained, washed twice with sterile phosphate-buffered saline and stored at −80 °C until use. Total RNA isolation was carried out using Trizol LS reagent (Gibco Life Sciences, BRL, Grand Island, NY, USA), according to the manufacturer's instructions. All RNA preparations and handling steps were conducted in a laminar flow hood, under RNAse-free conditions. Isolated RNA was dissolved in diethylpyrocarbonate-treated water and stored at −80 °C until use. RNA concentration was determined by absorbance reading at 260 nm using the Hitachi UV–VIS (U-2000) spectrophotometer (Tokyo, Japan). RNA integrity was tested by PCR amplification of the *β*-actin housekeeping gene. RNA isolated from MCF-7 breast cancer and the ARH-77 leukaemic cell lines was used as positive and negative controls, respectively.

Reverse transcription of RNA was carried out using the Thermoscript RT-PCR system (Invitrogen, Paisley, UK). cDNA was synthesised according to the manufacturer's instructions. The real-time RT–PCR assay for the detection of CK-19 mRNA-positive cells has already been described in detail (Stathopoulou *et al*, 2003). In brief, 2 *μ*l of cDNA was placed into 18 *μ*l of reaction volume containing 1 *μ*l of the sense primer CK-19-for (3 mmol l^−1^), 1 *μ*l of the anti-sense primer CK-19-do (3 mmol l^−1^), 2.4 *μ*l of the LightCycler-FastStart DNA Master Hybridization probes reagent (Roche, Mannheim, Germany) (10 × concentration), 1 *μ*l of the probe CK-19-FL (3 mmol l^−1^), and 1 *μ*l of the probe CK-19-LC (3 mmol l^−1^). The primers used have been described earlier (Stathopoulou *et al*, 2003). PCR was initiated with a 10-min denaturation at 95 °C and terminated with a 30-s cooling step at 40 °C. The cycling protocol consisted of a denaturation step at 95 °C, annealing at 60 °C for 10 s, and extension at 72 °C, and repeated 50 times. Fluorescence detection was performed at the end of each annealing step for <1 s. Real-time PCR for the housekeeping gene GAPDH was performed in all of the clinical samples to evaluate the quality of the cDNAs used in the study. The lower detection limit of the assay was set at 0.6 MCF-7 cell equivalents per 5 *μ*g of RNA (Stathopoulou *et al*, 2003). The within-run curve values (CVs) for MCF-7 cells as determined by the calibration curve ranged from 7.5 to 9.3%, whereas the corresponding crossing point (*C*p) values ranged from 0.9 to 1.5%; similarly, the between-run CVs ranged from 10.7 to 16.0%, whereas the corresponding *C*p values ranged from 2.2 to 3.2%. According to the analytic detection limit of our assay, the presence of ⩾0.6 MCF-7 equivalents per 5 *μ*g of total RNA derived from peripheral blood mononuclear cells was considered as a positive result (Stathopoulou *et al*, 2003). Using the above cutoff values, only 2 out of 89 healthy female donors were positive (2.2%) (Stathopoulou *et al*, 2003). Furthermore, none of the nine women with benign breast disease had positive blood samples. Twenty-seven bone marrow aspirates from normal donors (*n*=7) or patients with non-epithelial (*n*=5) or haematological malignancy (*n*=15) served as controls for the determination of the cutoff point for CK-19 mRNA expression in the bone marrow. The cutoff value, based on the 95th percentile of the CK-19 mRNA values of controls, was 0.6 MCF-7 equivalents per 5 *μ*g of total RNA.

### Study design

This is a retrospective analysis of prospectively collected data in the context of an ongoing longitudinal study. The aim of this study was to evaluate the detection rate and the prognostic relevance of the presence of CTCs and DTCs in patients with early breast cancer (McShane *et al*, 2005). Patients were enrolled during the period from March 1999 to January 2004. All patients signed an informed consent, which was approved by the Ethics and Scientific Committees of our Institution. As of June 2008, median follow-up time was 59.0 months (range, 13–95). Survival intervals were calculated from the first dose of chemotherapy until the date of death or of the first clinical or imaging evidence of disease recurrence.

### Statistical analysis

Differences of rates between groups were compared with either the two-sided Fisher's exact test or Pearson's *χ*^2^-test. Differences between groups in terms of continuous variables were assessed by the non-parametric Mann–Whitney test. The normality of continuous variables was verified using the Kolmogorov–Smirnov test. The Spearman exact test was performed to evaluate the correlation between the expression of CK-19 mRNA cells in the peripheral blood and bone marrow (Cox, 1970; Collett, 2003).

Differences in positivity rates between blood and bone marrow samples were assessed using the McNemar test. The Kaplan–Meier method was used to estimate DFS and survival curves, and the log-rank test was used to compare the curves (Altman, 1991; Armitage and Berry, 1994). The Cox proportional hazards model (Armitage and Berry, 1994) was used for outcomes related to time-to-event data and prognostic values. In order to construct such a model using covariates that have an independent significant influence on outcomes, a stepwise procedure (unconditional backward) was carried out (Armitage and Berry, 1994). Before the application of these methods, univariate analysis was performed for the preliminary exploration of variables of interest. *P*-values <0.05 were considered statistically significant for all comparisons.

## Results

### Patient characteristics

Patient characteristics are listed in Table 1. Patients' median age was 54 years (range, 26–75 years), 78 (47.3%) were pre-menopausal, (111) 91.5% had tumours >2 cm, 64 (38.8%) were of histological grade 3, 64 (38.8%) were ER negative, 33 (20%) were HER2 positive, and 106 (64.3) had infiltrated axillary lymph nodes.

### Detection of CTCs and DTCs before the initiation of adjuvant treatment

**Figure 1:**
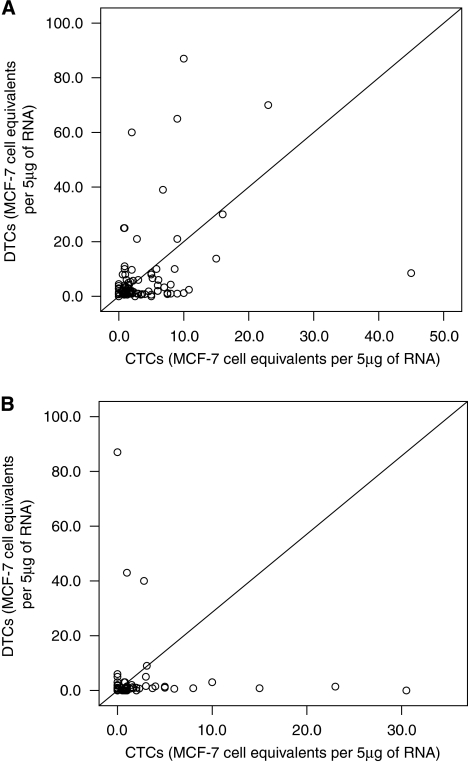
Correlation between the presence of CTCs and DTCs before initiation (**A**) and after the completion (**B**) of adjuvant chemotherapy.

CK-19 mRNA-positive CTCs and DTCs were detected in 91 (55.2%) and 95 (57.6%) of 165 patients, respectively. No correlation was found between the detection of CK-19 mRNA-positive CTCs or DTCs and the patients' clinical or pathological characteristics (Table 1). The median number of CTCs and DTCs was 2.0 (range, 0.6–149) and 2.0 (range, 0.6–732) MCF-7 equivalents per 5 *μ*g RNA, respectively (*P*=0.743). A strong correlation was observed between the detection of CK-19 mRNA-positive cells in peripheral blood and in bone marrow (Spearman test, *R*s=0.750; *P*=0.0001; Figure 1A).

As indicated in Table 2, 88 (53.3%) patients with detectable DTCs also had detectable CTCs (DTC(+)/CTC(+) group); in addition, in 67 (40.6%) patients, no occult tumour cells could be detected either in the bone marrow or peripheral blood (DTC(−)/CTC(−) group). In 3 (1.8%) patients with detectable CTCs, no DTCs were detected (DTC(−)/CTC(+) group), whereas in 7 (4.2%) patients with DTCs, no CTCs were detected (DTC(+)/CTC(−) group). Overall, there was 93.9% concordance (in 155 out of 165 patients) for the presence of occult tumour cells between peripheral blood and bone marrow, before the initiation of adjuvant chemotherapy. According to the McNemar test, the difference in positivity rates between the peripheral blood and bone marrow CK-19 mRNA expression was not statistically significant (*P*=0.344).

### Detection of CTCs and DTCs after the completion of adjuvant chemotherapy

In 162 patients, repeated blood samples were available after the completion of adjuvant chemotherapy. The CK-19 mRNA expression was evident in 79 (48.8%) patients compared with 90 (55.6%) patients before chemotherapy (*P*=0.169). Of 91 patients with detectable CK-19 mRNA-positive CTCs before chemotherapy, 58 (63.7%) remained positive and 32 (35.2%) were negative for CK-19 mRNA expression after chemotherapy (no data available for 1 patient). The median number of CTCs identified after chemotherapy was 1.3 (range, 0.6–25.0) equivalents per 5 *μ*g of RNA, which was not significantly different compared with pre-chemotherapy values (*P*=0.341). Conversely, 21 (28.4%) of 74 patients without detectable CK-19 mRNA-positive CTCs before chemotherapy became CK-19 mRNA positive after chemotherapy.

In the group of 84 patients with repeated bone marrow samples available after chemotherapy, 43 (51.2%) had CK-19 mRNA-positive DTCs compared with 53 (63.1%) patients before chemotherapy (*P*=0.099). In 33 (62.2%) of 53 initially DTC(+) patients, occult tumour cells persisted, whereas in 20 (37.7%), no tumour cells could be identified after chemotherapy. The median number of DTCs identified after chemotherapy was 1.0 MCF-7 cell equivalents per 5 *μ*g of RNA (range 0.6–87), which was significantly different compared with pre-chemotherapy values (*P*=0.045). Conversely, among 31 DTC(−) patients before chemotherapy, 10 (32.2%) had CK-19 mRNA-positive CTCs identified after chemotherapy.

A strong correlation was observed between the presence of CK-19 mRNA-positive CTCs and DTCs after chemotherapy (Spearman test, *R*s=0,696; *P*=0.0001, Figure 1B). Indeed, as indicated in Table 2, 44 (52.4%) out of 84 patients had detectable CTCs in peripheral blood after chemotherapy. A total of 32 (38.1%) patients with detectable DTCs also had detectable CTCs and 29 (34.5%) patients with a CK-19 mRNA-negative bone marrow also presented with a CK-19 mRNA-negative peripheral blood sample. The concordance rate for the bone marrow and peripheral blood CK-19 mRNA expression after the completion of adjuvant chemotherapy was 72.6%. According to the McNemar test, the difference in positivity rates between the blood and bone marrow CK-19 mRNA expression was not statistically significant (*P*=0.999).

With regard to the concomitant detection of tumour cells in the peripheral blood and bone marrow in the same group of 84 patients, 9 (18.4%) of the 49 DTC(+)/CTC(+) patients before chemotherapy were found negative in both peripheral blood and bone marrow, whereas 4 (13.7%) of 29 patients who were initially DTC(−)/CTC(−) became DTC(+)/CTC(+). Moreover, 13 (26.5%) of the initially DTC(+)/CTC(+) patients, as well as 8 (27.5%) of the initially DTC(−)/CTC(−) patients, had detectable occult tumour cells either in the bone marrow or peripheral blood after completion of adjuvant chemotherapy.

### Clinical relapses and disease-related deaths according to the detection of CK-19 mRNA-positive CTCs and DTCs

After a median follow-up period of 59.0 months (range, 13–95), 37 (22.4%) patients presented a local and/or distant relapse and 20 (12.1%) died because of disease progression. In the group of patients with CK-19 mRNA-positive CTCs (CTC(+) group) before the initiation of chemotherapy, 25 (27.5%) out of 91 relapsed, compared with 12 (16.2%) out of 74 patients in the CTC(−) group (Table 3; *P*=0.081). Among the 95 women with CK-19 mRNA-positive DTCs (DTC(+) group), 26 (27.4%) relapsed compared with 11 (15.7%) among 70 patients in the DTC(−) group (Table 3; *P*=0.105).

**Figure 2:**
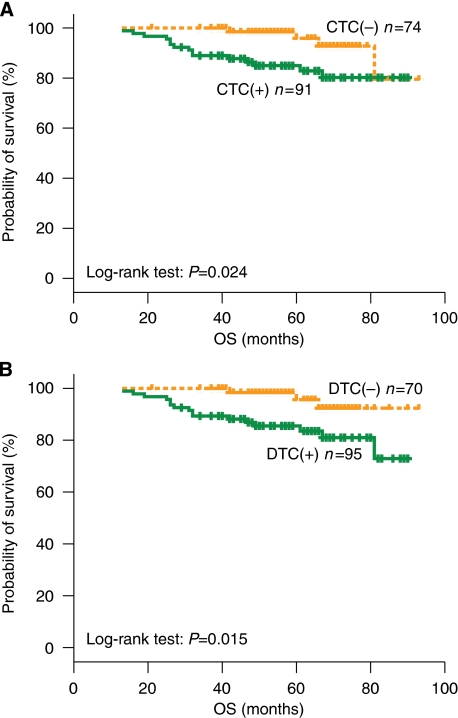
Overall survival curves of patients with early breast cancer according to the detection of CTCs (**A**) and DTCs (**B**) before chemotherapy.

When analysing survival data according to CTC status before chemotherapy, 15 (16.5%) out of 91 CTC(+) patients died compared with 5 (6.8%) out of 74 CTC(−) patients (Table 3). The Kaplan–Meier survival analysis showed a significantly reduced probability for OS for patients in the CTC(+) group compared with that in the CTC(−) group (log-rank test, *P*=0.024; Figure 2A). Similar results were observed for OS according to DTC status before chemotherapy. Specifically, 17 (17.9%) out of 95 patients in the DTC(+) group died compared with 3 (4.3%) out of 70 patients in the DTC(−) group (log-rank test, *P*=0.015; Figure 2B).

In the group of 162 patients with peripheral blood samples available after the completion of therapy, 22 (27.8%) out of 79 and 15 (18.1%) out of 83 relapsed in CTC(+) and CTC(−) groups, respectively (Table 3; *P*=0.057). Among the group of 84 patients with a bone marrow sample obtained after chemotherapy, the number of relapses was 11 (25.6%) out of 43 and 6 (14.6%) out of 41 patients (*P*=0.162) in the DTC(+) and DTC(−) groups, respectively. No significant difference was observed in patients' survival according to the detection of CTCs (*P*=0.128) or DTCs (*P*=0.302) after chemotherapy (Table 3).

### Clinical outcome according to the concomitant detection of CK-19 mRNA-positive CTCs and DTCs

**Figure 3:**
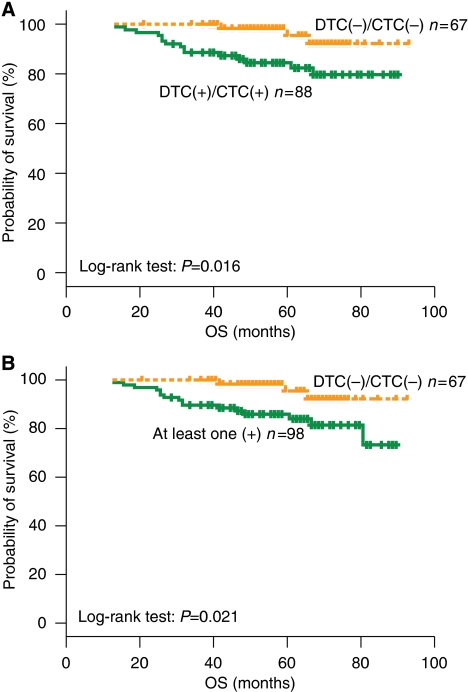
Overall survival curves of patients with early breast cancer according to the concomitant detection of CTCs and DTCs. (**A**) Both positive *vs* both negative. (**B**) Both negative *vs* at least one positive (‘other combinations’).

The outcome of patients according to the concomitant detection of CK-19 mRNA-positive CTCs and DTCs before the initiation of chemotherapy is presented in Table 4. The incidence of relapses was 27.3 and 14.9% in patients of DTC(+)/CTC(+) and DTC(−)/CTC(−) groups before chemotherapy, respectively (Table 4; *P*=0.066); 3 (30%) additional relapses occurred among 10 patients who had detectable DTCs or CTCs (group ‘other combinations’; Table 4). A total of 15 (17%) deaths occurred in patients of the DTC(+)/CTC(+) group compared with 3 (4.5%) in the subgroup of patients with a DTC(−)/CTC(−) status before chemotherapy. The Kaplan–Meier survival analysis showed a significantly longer OS in patients of the DTC(−)/CTC(−) group compared with those of the DTC(+)/CTC(+) group (log-rank test, *P*=0.016; Figure 3A). Moreover, OS was significantly longer in patients with a DTC(−)/CTC(−) status compared with those with at least one positive result designated as ‘other combinations’ (log-rank test, *P*=0.021; Figure 3B). A total of 6 (22.1%) deaths occurred in the group of patients who had persistently positive peripheral blood and bone marrow, both before and after chemotherapy; conversely, no deaths were observed in patients without detectable CK-19 mRNA-positive CTCs and DTCs, either before or after the completion of chemotherapy (log-rank test, *P*=0.036).

### Univariate and multivariate analysis

As shown in Table 5, tumour size, histological grade and ⩾4 involved axillary lymph nodes were significantly associated with decreased DFS in univariate analysis. Factors associated with decreased OS were the detection of CTCs, DTCs, as well as the simultaneous detection of CTCs and DTCs before chemotherapy.

A multivariate analysis including factors that were most significantly associated with DFS and OS in the univariate analysis showed that tumour size (*P*=0.031) and the involvement of ⩾4 axillary lymph nodes (*P*=0.036) were independent factors associated with decreased DFS (Table 6), whereas histological grade (*P*=0.022), PR negativity (*P*=0.013), and the detection of DTCs before chemotherapy (*P*=0.032) were independently associated with decreased OS (Table 6). Owing to the significant correlation observed between the detection of CTCs and DTCs, the CTC status before chemotherapy was not included in the multivariate analysis.

## Discussion

The data presented in this study show that there is a significant concordance between the detection of CK-19 mRNA-positive cells detected in the peripheral blood (CTCs) and in bone marrow (DTCs) of patients with early breast cancer, both before initiation and after completion of adjuvant chemotherapy. In addition, it was shown that the detection of CTCs or DTCs before chemotherapy was associated with decreased survival of patients.

Using a quantitative RT-PCR for detecting CK-19 mRNA-positive cells, we observed a concordance rate of 93.9 and 72.6% between peripheral blood and bone marrow samples obtained before initiation and after completion of adjuvant chemotherapy, respectively, in patients with early breast cancer. Similar observations have been reported by other investigators who evaluated the presence of CK-positive cells in the peripheral blood and bone marrow using immunocytochemical assays (Witzig *et al*, 2002; Pierga *et al*, 2004; Muller *et al*, 2005; Wiedswang *et al*, 2006). In a previous report from our group, we reported a weaker correlation between the detection of CK-19 mRNA expression in blood and in the bone marrow in a smaller series of patients with early breast cancer using a qualitative nested RT-PCR assay (Stathopoulou *et al*, 2002). Differences in the assays used could account for these discrepancies. Indeed, Schoenfeld *et al* (1997) have reported that the concordance between blood and bone marrow samples for occult tumour cell detection was only 6% by immunocytochemistry and 27% by RT-PCR.

The lack of a complete overlap between the presence of CK-19 mRNA-positive CTCs and DTCs either before (Ismail *et al*, 2004; Pierga *et al*, 2004; Muller *et al*, 2005; Wiedswang *et al*, 2006) or after chemotherapy (Slade *et al*, 2009) could be related to the fact that occult tumour cells are rare events and therefore their evaluation is greatly influenced by sampling variability. In addition, it has been suggested that blood represents a temporary compartment for disseminated cells (Muller *et al*, 2005), whereas only a subpopulation of CTCs can settle in distant organs such as the bone marrow (Muller *et al*, 2005). Nevertheless, the high concordance observed between CTC and DTC detection in our study favours the hypothesis that there is a continuous circulation and exchange of epithelial cells between bone marrow and peripheral blood.

The high detection rate of occult tumour cells in both peripheral blood and bone marrow observed in the current study is in overt contrast with previous studies reporting low detection rates using immunocytochemical assays (Ismail *et al*, 2004; Pierga *et al*, 2004; Muller *et al*, 2005; Wiedswang *et al*, 2006; Benoy *et al*, 2006) and should be attributed to the high sensitivity and specificity of the quantitative RT-PCR assay used for the detection of minimal residual disease (Stathopoulou *et al*, 2003). Moreover, it is interesting to note that only 10% of 1767 patients with early disease had more than one CTC per 23 ml of peripheral blood when tested with the new automated Cell Search System (Rack *et al*, 2007, 2008).

This analysis also shows that CK-19 mRNA-positive tumour cells persist in the blood and/or bone marrow of patients with early breast cancer despite the administration of adjuvant chemotherapy. Indeed, 63.7 and 62.2% of patients in the CTC(+) and DTC(+) groups before the initiation of chemotherapy, respectively, had occult tumour cells detected in their blood or bone marrow after chemotherapy. In addition, 28.4 and 32.2% of patients without detectable CTCs or DTCs, before chemotherapy, respectively, presented with CK-19 mRNA-positive cells after the completion of adjuvant chemotherapy. This observation suggests that chemoresistant cells in patients identified as DTC(−) or/and CTC(−) because of a low tumour load that is undetectable by the assay could proliferate during the administration of adjuvant chemotherapy and reach the detection limit after the end of treatment. Moreover, the increase in discordance between the detection of CTCs and DTCs after chemotherapy suggests that CTCs and DTCs represent biologically and genetically different cells with a variable sensitivity to chemotherapy.

The above observations point to the presence of subpopulations of CTCs or/and DTCs that bear an inherent resistance to the chemotherapy regimen used as adjuvant treatment. The presence of chemoresistant CTCs or DTCs has been previously described (Braun *et al*, 2000a; Xenidis *et al*, 2003, 2009) and attributed to the low proliferative capacity of these cells (Pantel *et al*, 1993; Muller *et al*, 2005). Therefore, it could be hypothesised that the detection of occult tumour cells after the completion of adjuvant chemotherapy could be used as a surrogate marker for the efficacy of the adjuvant treatment used.

Previous reports evaluating the prognostic significance of disseminated tumour cells in blood and bone marrow concluded that the detection of CTCs is prognostically inferior to the detection of DTCs (Pierga *et al*, 2004; Benoy *et al*, 2006; Wiedswang *et al*, 2006). In this study, the presence of CK-19 mRNA-positive CTCs and DTCs before the initiation of adjuvant chemotherapy was predictive for decreased OS. Moreover, in the multivariate analysis, the detection of DTCs was an independent prognostic factor associated with a significantly higher risk of death.

Several reports have shown that the persistence of occult tumour cells in the bone marrow (Wiedswang *et al*, 2004; Janni *et al*, 2005) or peripheral blood (Xenidis *et al*, 2003, 2007, 2009) after adjuvant chemotherapy or during follow-up is associated with an adverse clinical outcome. In this study, the detection of CTCs and/or DTCs after chemotherapy was not associated with increased risk for relapse or death. Further prospective studies including a larger number of patients are needed to clarify which is the most relevant time point for the evaluation of minimal residual disease before or after adjuvant chemotherapy. Moreover, the reproducibility and stability of the assays used should also be evaluated in a routine clinical setting.

In conclusion, the results of this study indicate that the detection of CK-19 mRNA-positive DTCs and CTCs both before and after chemotherapy is correlated in patients with early-stage breast cancer. The determination of the CTC status was shown to convey prognostic information that was of comparable clinical relevance with that of DTC status. However, for convenience, the detection of CTCs could be used as an alternative to bone marrow for the identification of occult breast cancer cells and for monitoring minimal residual disease. This is of particular interest in the light of the results of recent trials, indicating that sequential treatment during the disease-free period may improve OS in breast cancer (Coombes *et al*, 2007; Kaufmann *et al*, 2007). Conceivably, secondary adjuvant treatment could be administered in patients selected on the basis of persisting CTCs. However, this should be tested in prospective clinical trials.

## Figures and Tables

**Table 1:** Patients' characteristics

	**PB**				**BM**		
		**CK-19+**	**CK-19−**	***P*-value**	**CK-19+**	**CK-19−**	***P*-value**
**Patients enrolled (*n*)**	**165**	**91**	**74**		**95**	**70**	
*Age (years)*							
Median	54	54	54.5	0.647	54	53	0.877
Range	26–75	26–74	36–75		26–74	36–75	
							
	*n* (%)	*n* (%)			*n* (%)		
*Menopausal status*							
Pre-menopausal	78 (47.3)	45 (49.5)	33 (44.6)	0.534	44 (46.3)	34 (48.6)	0.774
Post-menopausal	87 (52.7)	46 (50.5)	41 (55.4)		51 (53.7)	36 (51.4)	
							
*Tumour size (cm)*							
<2.0	54 (32.7)	27 (29.7)	27 (36.5)	0.650	29 (30.5)	25 (35.7)	0.768
2.0–5.0	97 (58.8)	56 (61.5)	41 (55.4)		58 (61.1)	39 (55.7)	
>5.0	14 (8.5)	8 (8.8)	6 (8.1)		8 (8.4)	6 (8.6)	
							
*Lymph nodes*							
0	59 (35.8)	28 (30.8)	31 (41.9)	0.240	30 (31.6)	29 (41.4)	0.294
1–3	59 (35.8)	33 (36.3)	26 (35.1)		34 (35.8)	25 (35.7)	
⩾4	47 (28.5)	30 (33.0)	17 (23.0)		31 (32.6)	16 (22.9)	
							
*Histology grade*							
I/II	79 (47.9)	41 (45.1)	38 (51.4)	0.669	44 (46.3)	35 (50.0)	0.800
III	64 (38.8)	38 (41.8)	26 (35.1)		37 (38.9)	27 (38.6)	
Lobular^a^	22 (13.3)	12 (13.2)	10 (13.5)		14 (14.7)	8 (11.4)	
							
*ER status*							
ER^+^	101 (61.2)	59 (64.8)	42 (56.8)	0.290	60 (63.2)	41 (58.6)	0.550
ER^−^	64 (38.8)	32 (35.2)	32 (43.2)		35 (36.8)	29 (41.4)	
							
*PR status*							
PR^+^	88 (53.3)	50 (54.9)	38 (51.4)	0.645	50 (52.6)	38 (54.3)	0.833
PR^−^	77 (46.7)	41 (45.1)	36 (48.6)		45 (47.4)	32 (45.7)	
							
*HER2 status (ICH)*							
Negative (0–2)	131 (79.4)	78 (85.7)	58 (78.4)	0.290	82 (86.3)	54 (77.1)	0.176
Positive (3+)	33 (20.0)	13 (14.3)	15 (20.3)		13 (13.7)	15 (21.4)	
UN	1 (0.6)		1 (1.3)			1 (1.4)	
							
*Surgery*							
S	119 (72.1)	62 (68.1)	57 (77.0)	0.205	64 (67.4)	55 (78.6)	0.113
T	46 (27.9)	29 (31.9)	17 (23.0)		31 (32.6)	15 (21.4)	
							
*Adjuvant chemotherapy*							
FEC	60 (36.4)	37 (40.7)	23 (31.1)	0.055	37 (38.9)	23 (32.9)	0.055
T/EC	98 (59.4)	53 (58.2)	45 (60.8)		57 (60.0)	41 (58.6)	
CMF	7 (4.2)	1 (1.1)	6 (8.1)		1 (1.1)	6 (8.6)	
							
*RT*							
Yes	144 (87.3)	83 (91.2)	61 (82.4)	0.093	85 (89.5)	59 (84.3)	0.323
No	21 (12.7)	8 (8.8)	13 (17.6)		10 (10.5)	11 (15.7)	

BM=bone marrow; CK-19=cytokeratin-19; ER=oestrogen receptor; PR=progesterone receptor; PB=peripheral blood; RT=radiotherapy; S=segmentectomy/lumpectomy; T=total radical mastectomy; UN=unknown.

a Lobular carcinomas (*n*=22) are not scored by histological grade.

**Table 2:** Concordance of CTCs and DTCs

**Pre-chemotherapy (*n*=165)**			**Post-chemotherapy (*n*=84)**		
***n***	**CTC(+) 91 (55.2%)**	**CTC(−) 74 (44.8%)**	***n***	**CTC(+) 44 (52.4%)**	**CTC(−) 40 (47.6%)**
DTC(+) 95 (57.6%)	88 (53.3%)	7 (4.2%)	DTC (+) 43 (51.2%)	32 (38.1%)	11 (13.1%)
DTC(−) 70 (42.4%)	3 (1.8%)	67 (40.6%)	DTC (−) 41 (48.8%)	12 (14.2%)	29 (34.5%)

CTC=circulating tumour cell; DTC=disseminated tumour cell.

**Table 3:** Incidence of relapses and disease-related deaths according to the detection of CK-19 mRNA(+) CTCs and DTCs as considered separately

		**Relapse *n* (%)**	***P*-value**	**Death *n* (%)**	***P*-value**
*Pre-chemotherapy*					
CTC status (*n*=165)	CTC(+) (*n*=91)	25 (27.5%)		15 (16.5%)	
	CTC(−) (*n*=74)	12 (16.2)	0.081	5 (6.8)	**0.024**
					
DTC status (*n*=165)	DTC(+) (*n*=95)	26 (27.4)		17 (17.9)	
	DTC(−) (*n*=70)	11 (15.7)	0.105	3 (4.3)	**0.015**
					
*Post-chemotherapy*					
CTC status (*n*=162)	CTC(+) (*n*=79)	22 (27.8)		12 (15.2)	
	CTC(−) (*n*=83)	15 (18.1)	0.057	8 (9.6)	0.128
					
DTC status (*n*=84)	DTC(+) (*n*=43)	11 (25.6)		7 (16.3)	
	DTC(−) (*n*=41)	6 (14.6)	0.162	5 (12.2)	0.302

CK-19=cytokeratin-19; CTC=circulating tumour cell; DTC=disseminated tumour cell.

Significant *P*-values are shown in bold.

**Table 4:** Incidence of relapses and disease-related deaths according to the concomitant detection of CK-19 mRNA(+) CTCs and DTCs pre-chemotherapy

**DTC/CTC status**	**Clinical relapses**			**Deaths**		
	**Yes**	**No**	***P*-value**	**Yes**	**No**	***P*-value**
CTC(+)/DTC(+) (*n*=88)	24 (27.3%)	64 (72.7%)		15 (17.0%)	73 (83.0%)	
CTC(−)/DTC(−) (*n*=67)	10 (14.9%)	57 (85.1%)	0.066	3 (4.5%)	64 (95.5%)	**0.016**
Other combinations^a^ (*n*=10)	3 (30.0%)	7 (70.0%)		2 (20.0%)	16 (80.0%)	

CK-19=cytokeratin-19; CTC=circulating tumour cell; DTC=disseminated tumour cell.

a (CTC(+)/DTC(−) or CTC(−)/DTC(+)).

Significant *P*-values are shown in bold.

**Table 5:** Predictive and prognostic factors by univariate analyses (unadjusted relative risk) for DFS and OS

	**Log-rank test**	***P*-value**	**Hazard ratio**	***P*-value**	**95% CI**
*DFS*					
CTC (+ *vs* −) apre- (*n*=165)	3.042	0.081	1.827	0.087	0.917–3.641
DTC (+ *vs* −) pre- (*n*=165)	2.621	0.105	1.775	0.111	0.876–3.597
CTC (+ *vs* −) apost- (*n*=162)	3.613	0.057	1.873	0.062	0.969–3.621
DTC (+ *vs* −) post- (*n*=84)	1.960	0.162	2.008	0.171	0.741–5.441
DTC+/CTC+ *vs* DTC−/CTC− pre- (*n*=155)	3.189	0.074	1.933	0.080	0.924–4.046
DTC/CTC at least one (+) *vs* both (−) pre-(*n*=165)	3.170	0.075	1.910	0.081	0.924–3.949
DTC/CTC at least one (+) *vs* both (−) post-(*n*=84)	1.891	0.169	2.162	0.180	0.701–6.668
Menopausal status (post *vs* pre) (*n*=165)	3.300	0.069	1.851	0.074	0.941–3.641
Tumour size (>2 *vs* ⩽2 cm) (*n*=165)	6.495	**0.011**	2.655	**0.015**	1.213–5.809
Grade (III *vs* I/II) (*n*=143)	6.969	**0.008**	2.435	**0.011**	1.229–4.824
Nodal involvement (⩾4 *vs* 0–3) (*n*=165)	6.525	**0.011**	2.275	**0.013**	1.188–4.357
ER (− *vs* +) (*n*=165)	1.182	0.277	1.428	0.281	0.747–2.727
PR (− *vs* +) (*n*=165)	3.349	0.067	1.829	0.072	0.947–3.531
Adjuvant CMT (Other *vs* T/EC) (*n*=165)	2.260	0.133	1.636	0.138	0.854–3.134
					
*OS*					
CTC (+ *vs* −) pre- (*n*=165)	5.125	**0.024**	3.319	**0.033**	1.101–10.004
DTC (+ *vs* −) pre- (*n*=165)	5.866	**0.015**	4.074	**0.026**	1.187–13.990
CTC (+ *vs* −) post- (*n*=162)	2.311	0.128	2.034	0.137	0.798–5.183
DTC (+ *vs* −) post- (*n*=84)	1.063	0.302	1.889	0.311	0.552–6.463
DTC+/CTC+ *vs* DTC−/CTC− pre-(*n*=155)	5.857	**0.016**	4.094	**0.026**	1.185–14.144
DTC/CTC at least one (+) *vs* both (−) pre-(*n*=165)	5.309	**0.021**	3.839	**0.033**	1.118–13.180
DTC/CTC at least one (+) *vs* both (−) post-(*n*=84)	2.173	0.140	3.002	0.161	0.647–13.943
Menopausal status (post- *vs* pre-) (*n*=165)	1.210	0.271	1.678	0.277	0.660–4.269
Tumour size (>2 *vs* ⩽2 cm) (*n*=165)	4.884	**0.027**	3.657	**0.039**	1.065–12.552
Grade (III *vs* I/II) (*n*=143)	7.587	**0.006**	3.653	**0.010**	1.366–9.770
Nodal involvement (⩾4 *vs* 0–3) (*n*=165)	3.743	0.053	2.379	**0.061**	0.961–5.890
ER (− *vs* +) (*n*=165)	5.115	**0.024**	2.798	**0.031**	1.101–7.110
PR (− *vs* +) (*n*=165)	8.712	**0.003**	4.549	**0.007**	1.507–13.734
Adjuvant CMT (T/EC *vs* other) (*n*=165)	0.210	0.729	1.174	0.729	0.474–2.903

−=negative; +=positive; CI=confidence interval; CTC=circulating tumour cell; DFS=disease-free survival; DTC=disseminated tumour cell; ER=oestrogen receptor; OS=overall survival; PR=progesterone receptor.

a pre- and post- refer to the pre- and post-chemotherapy values CTC and DTC.

Significant *P*-values are shown in bold.

**Table 6:** Multivariate analysis for DFS and OS

**Parameter**	**Hazard ratio**	**95% CI**	***P*-value**
*DFS*			
Tumour size (>2 *vs* ⩽2 cm)	2.391	1.085–5.273	**0.031**
Lymph nodes (⩾4 *vs* 0–3)	2.014	1.045–3.881	**0.036**
			
*OS*			
Histology grade (III *vs* I/II)	3.220	1.184–8.762	**0.022**
PR (− *vs* +)	4.061	1.337–12.332	**0.013**
DTC (+ *vs* −) pre-chemotherapy	3.859	0.119–13.303	**0.032**

CI=confidence interval; DFS=disease-free survival; DTC=disseminated tumour cell; OS=overall survival; PR=progesterone receptor.

Significant *P*-values are shown in bold.
